# Expectant fathers’ participation in antenatal care services in Papua New Guinea: a qualitative inquiry

**DOI:** 10.1186/s12884-018-1759-4

**Published:** 2018-05-08

**Authors:** Jessica Davis, Cathy Vaughan, Justine Nankinga, Lisa Davidson, Hellen Kigodi, Eileen Alalo, Liz Comrie-Thomson, Stanley Luchters

**Affiliations:** 1 Burnet Institute, Melbourne, Victoria, Australia; 2 Melbourne School of Population and Global Health, University of Melbourne, Victoria, Australia; 3 United Nations Children’s Fund (UNICEF), Papua New Guinea Country Office, Port Moresby, Papua New Guinea; 4 current FHI 360, Port Moresby, Papua New Guinea; 5 Department of Epidemiology and Preventive Medicine, Faculty of Medicine Nursing and Health Science, Monash University, Melbourne, Australia; 6 Catholic Church Health Services, Mingende, Port Moresby, Papua New Guinea; 7 International Centre for Reproductive Health, Department of Obstetrics and Gynecology, Faculty of Medicine and Health Sciences, Ghent University, Ghent, Belgium

**Keywords:** Antenatal, Maternal health, Engaging men, Male involvement, Qualitative, Papua New Guinea

## Abstract

**Background:**

The importance of engaging men in maternal and child health programs is well recognised internationally. In Papua New Guinea (PNG), men’s involvement in maternal and child health services remains limited and barriers and enablers to involving fathers in antenatal care have not been well studied. The purpose of this paper is to explore attitudes to expectant fathers participating in antenatal care, and to identify barriers and enablers to men‘s participation in antenatal care with their pregnant partner in PNG.

**Methods:**

Twenty-eight focus group discussions were conducted with purposively selected pregnant women, expectant fathers, older men and older women across four provinces of PNG. Fourteen key informant interviews were also conducted with health workers. Qualitative data generated were analysed thematically.

**Results:**

While some men accompany their pregnant partners to the antenatal clinic and wait outside, very few men participate in antenatal consultations. Factors supporting fathers’ participation in antenatal consultations included feelings of shared responsibility for the unborn child, concern for the mother’s or baby’s health, the child being a first child, friendly health workers, and male health workers. Sociocultural norms and taboos were the most significant barrier to fathers’ participation in antenatal care, contributing to men feeling ashamed or embarrassed to attend clinic with their partner. Other barriers to men’s participation included fear of HIV or sexually transmitted infection testing, lack of separate waiting spaces for men, rude treatment by health workers, and being in a polygamous relationship. Building community awareness of the benefits of fathers participating in maternal and child health service, inviting fathers to attend antenatal care if their pregnant partner would like them to, and ensuring clinic spaces and staff are welcoming to men were strategies suggested for increasing fathers’ participation in antenatal care.

**Conclusion:**

This study identified significant sociocultural and health service barriers to expectant fathers’ participation in antenatal care in PNG. Our findings highlight the need to address these barriers – through health staff training and support, changes to health facility layout and community awareness raising – so that couples in PNG can access the benefits of men’s participation in antenatal care.

**Electronic supplementary material:**

The online version of this article (10.1186/s12884-018-1759-4) contains supplementary material, which is available to authorized users.

## Background

Globally, maternal and newborn mortality remains unacceptably high. Over three hundred thousand maternal deaths and close to 3 million newborn deaths occur annually, with the vast majority of deaths occurring in developing countries [1, 2]. Papua New Guinea (PNG), located in the Asia Pacific region, continues to experience high maternal and newborn mortality and morbidity. Current estimates of maternal mortality vary between 215 and 733 deaths per 100,000 live births [3–5]. The leading causes of maternal mortality – post-partum haemorrhage, eclampsia and sepsis – are similar to elsewhere in the world and largely preventable [6, 7]. Newborn mortality is also high at 25 per 1000 [8] and international estimates suggest that up to two thirds of these deaths could be prevented with effective, basic care [9].

The Government of PNG has identified early and ongoing antenatal care (ANC) and skilled care at birth as key strategies for addressing these poor health outcomes [5, 10]. Currently only 67% of pregnant women receive any ANC [11] and only 55% of pregnant women receive the recommended four or more ANC visits [8]. Further, less than half (44%) of women receive skilled care during childbirth [11]. Gender inequality is a significant issue in PNG; women and girls have significantly less access to health and education than men and boys, violence against women and girls is common, and inequitable decision-making in the home contributes to poor health outcomes [12, 13]. Women’s lack of decision-making power and lack of male partner support have been highlighted as important barriers to women’s use of health care services during pregnancy and for childbirth [13–16].

Global research suggests that involving expectant fathers in antenatal education, either through participation in ANC consultations with their pregnant partner or in antenatal education interventions, can be an effective strategy for improving health behaviours during pregnancy [17], increasing women’s utilisation of skilled childbirth care and postpartum care [18], and can be effective in increasing ANC attendance in some contexts [17, 19–23]. Engaging men can also contribute to improved couple communication and shared decision-making regarding MCH [24, 25], and is therefore particularly critical in settings such as PNG where gender inequality limits women’s access to services [15, 16]. Engaging men in ANC and prevention of parent-to-child transmission of HIV (PPTCT) initiatives has also demonstrated positive impacts on the proportion of pregnant women and couples testing for HIV, the use of condoms or abstinence to prevent HIV transmission within the couple, adherence to drug prophylaxis regimes and recommended infant feeding practices by HIV-positive mothers, and subsequently increase HIV-free infant survival [26–30].

Despite wide variation across different contexts, barriers to men’s involvement in MCH services commonly identified globally include beliefs that it is unnecessary or inappropriate for men to participate in pregnancy or postpartum care, or men feeling embarrassed or ashamed to participate in MCH services [31–40], and men not being invited to attend services [31]. Other common barriers include fear of being tested for sexually transmitted infections (STIs) and HIV at a clinic [40, 41], fear of being perceived as a jealous husband following his wife around [34] or fear of a man being perceived as ‘dominated’ by his female partner [33, 37]. Inappropriate opening hours or long waiting time [37, 39, 42], work commitments or low job security preventing men taking time off work [31, 41–43], negative health worker attitudes towards men’s involvement in MCH services and lack of staff capacity or space to engage men [31, 33, 35, 37, 39, 41, 44] have also been identified as barriers to engaging men in clinical settings. Poor understanding among men of the health problems faced by mothers and babies and inadequate knowledge regarding how to take an active role in MCH can also impede men’s participation in ANC and MCH [31, 33, 41, 44, 45]. Men’s involvement in MCH has also been negatively correlated with distance to the health facility [46], having multiple children [47], and women’s autonomy [48, 49], and positively correlated with wife’s education level [47], and male partner income and education [39].

Despite significant barriers in many contexts that act against men participating in antenatal care, research in diverse settings has shown that many men and women welcome greater men’s involvement in MCH services [31, 35, 41, 50–52], that many men want and need more information regarding women’s and children’s health [31, 35, 41, 53], and that a range of strategies can effectively increase men’s participation in ANC and men’s support for MCH [23, 35, 54–58].

The *World Health Organization’s Recommendations on Health Promotion Interventions for Maternal and Newborn Health* recommend interventions to engage fathers during pregnancy, childbirth and the postnatal period [19]. In PNG, the *National Sexual & Reproductive Health Policy* [59] explicitly advocates for the active involvement of expectant fathers in ANC and in the labour ward during childbirth. However, in practice most expectant fathers do not attend ANC consultations with their pregnant partner or participate in any formal antenatal education, and in many PNG communities pregnancy, birth and infant care are considered ‘women’s business’ resulting in limited men’s involvement in maternal and child health (MCH) more generally [41, 44]. Lack of engagement with expectant fathers during the antenatal period is a missed opportunity to deliver critical information and services that improve MCH, including STI and HIV testing, treatment and preventive education. The purpose of this paper is to explore barriers, enablers and potential strategies for involving expectant fathers in ANC with their pregnant partner in PNG.

## Methods

### Study design and purpose

This paper forms part of a larger study that the authors conducted between June and August 2012 to examine health seeking behaviour for antenatal care, men’s involvement in ANC, and prevention, testing and treatment of STIs and HIV. This study employed a qualitative study design including focus group discussions (FGDs) and key informant interviews (KIIs) using standard question guides. Given the relative dearth of published research into expectant fathers’ involvement in ANC in PNG, this study design was appropriate to explore and build our understanding of this topic. This study was funded by UNICEF PNG and conducted to inform design of the “*Haus Man Sambai Long Ol Mama”* project, a UNICEF PNG pilot program to increase men’s involvement in ANC and PPTCT.

### Study setting

Data were collected across four provinces of PNG. All four provinces had been identified as implementation locations for the *Haus Man Sambai Long Ol Mama* project, selected for the project due to high rates of parent-to-child transmission of HIV. In each province, one to three clinics providing PPTCT services were purposively selected to represent the range of rural and urban clinical settings operating in that province. Ultimately, a total of seven sites were selected: Port Moresby General Hospital and St. Therese Clinic in National Capital District; Migende St. Joseph Rural Hospital in Chimbu Province; Kumin Headquarters PPTCT Centre and Mendi General Hospital in Southern Highlands Province; and Mt. Hagen General Hospital, Tininga and Rabiamul Clinic in Western Highlands Province. Antenatal care services are provided free of charge in these clinic.

### Participants and sampling

The main study participants were adult men and women from communities surrounding the study clinics, including: women who were pregnant or had given birth in the last 12 months (referred to collectively here as ‘pregnant mothers’); men whose female partner was currently pregnant or had given birth in the last 12 months (referred to collectively here as ‘expectant fathers’); older women aged 50 years or over; and older men aged 50 years or over. In PNG, older people often play an important role in community decision-making and information sharing and older women in particular often support younger women throughout pregnancy and childbirth [44]. Older people were therefore an important informant group to understand attitudes and experiences of men’s role in the antenatal period. FGD participants were recruited using convenience sampling via public announcements, flyers, posters, and individual verbal invitations at health centres, community meeting places, churches and other community institutions.

A total of 300 community members participated in FGDs, including 78 pregnant mothers, 64 expectant fathers, 77 older women and 81 older men. The average pregnant mother participating in this study was 27 years old (range 17 to 45 years), with two or three children (range zero to 8) and 5.6 years of schooling (range zero to 16). The average expectant father was 31 years old (range 19 to 46 years), had two or three children (range zero to 10 children), and had 6.6 years of schooling (range zero to 12 years). Most pregnant mothers and expectant fathers lived with their partner (94% of mothers and 89% of fathers) and were unemployed (88% of mothers and 64% of fathers). A substantial proportion of all participants were in a polygamous marriage, including 13% of pregnant mothers and 5% of expectant fathers. Older men and women were all believed to be aged over 50 years, although many could not recall their exact age. Older women had an average of four children (range zero to eight) and older men an average of between five and six children (range zero to 15). The average older woman had 3.9 years of schooling (range zero to 14 years) while the average older man had 3.6 years of schooling (range zero to 12 years).

Health workers (nurses and midwives) involved in ANC or PPTCT service provision in local hospitals, health centres or clinics were also eligible to participate in KIIs. Two health workers were purposively recruited at each site by trained data collectors in conversation with health service management.

### Data collection

A total of 28 FGDs (four per site) were conducted. At each site, separate FGDs were conducted with pregnant mothers, expectant fathers, older men and older women. Trained FGD facilitators used open-ended question guides developed and pilot-tested for each specific participant group (refer to Additional files 1, 2, 3, 4, and 5). FGDs were led by a facilitator of the same gender as the participants, supported by a note taker, and explored attitudes to expectant fathers’ participation in ANC, and barriers, enablers and potential strategies to promote men’s participation in ANC. FGDs were held in private rooms in community buildings or health facilities, involved between four and 11 participants, lasted approximately 1.5 h and were conducted in *Tok Pisin* in combination with other local languages. Basic demographic data were collected from FGD participants regarding number of children, marital status and age.

We also undertook 14 KIIs (two per site) with health workers to explore attitudes and behaviours relevant to expectant fathers’ participation in ANC, health system factors influencing expectant fathers’ participation and opportunities to promote fathers’ participation in ANC. KIIs were conducted face-to-face in a private space in the health facility by one facilitator and one note taker, lasted approximately one hour and were conducted in *Tok Pisin* or English, depending on the participant’s preference.

This study used different data collection personnel in each province. Provincial teams were supervised by a team leader who managed and participated in data collection across all sites. This approach was adopted to develop research capacity in each location, to minimise security risks to study personnel, and to ensure data collectors were fluent in local languages. Provincial data collectors were community HIV educators sourced from local non-government organisations. Most had limited prior experience of qualitative research. All data collectors participated in a five day training workshop on qualitative research and participated in field-testing study tools.

Two digital recorders were used during data collection and data collectors took detailed written notes. However, substantial background noise and softly spoken participants compromised the usefulness of some digital recordings. Detailed notes taken by note-takers in each FGD and KII were compared to voice recordings and amended where possible and if required, before being checked by the FGD or KII facilitator. These written records were then translated into English.

### Data analysis

Translated written records were reviewed based on broad themes of interest, namely: support that fathers currently provide to pregnant partners; attitudes to fathers participating in ANC; barriers to expectant fathers attending ANC; enablers to fathers participating in ANC; and potential strategies for increasing fathers’ participation in ANC. Subsequent analysis of written records involved inductive data-driven coding of the text to identify and synthesise recurrent issues in the data. The software package NVivo 10 was used for data management. Provincial research teams provided feedback on initial interpretation of the data, the recurrent issues identified during analysis, and provided assistance with interpretation of findings at a two-day workshop in Port Moresby.

### Ethics, consent and permissions

This research was approved by the Research Advisory Council of the National AIDS Council Secretariat in Papua New Guinea and the Alfred Health Human Research Ethics Committee in Australia. Written or verbal consent to participate was obtained from all participants after data collectors had explained study objectives and procedures and checked that participants understood this information. To protect participant confidentiality, quotes are attributed to the participant group and the province, but without reference to individual sites. Because of the small number of health worker participants, health worker quotes are not attributed to a province.

## Results

### Expectant fathers’ support for their pregnant partner

FGD and KII participants in all provinces reported that some men accompany their partner to the ANC clinic and wait nearby, but that few men participate in ANC consultations with their partner. Participants report that many men support their pregnant partner in other ways, including providing nutritious food and helping with heavier chores such as carrying water, chopping firewood, gardening or other housework. Expectant fathers also commonly provide financial assistance to their pregnant partner, predominantly for bus fares, hospital fees or food, or play a role in organising transport for women to access the health facility. Some men also assist their pregnant partner by caring for older babies or children.

*Some men do help their wives, especially those men who stay at home with their wives. They help in terms of food and firewood and assist in caring for the baby when the baby is crying or, they even feed them [the baby].* (Pregnant Mothers’ FGD, Chimbu Province).

Some expectant fathers also provide emotional support and encouragement to their pregnant partner; by discussing health information or providing advice, by discussing her concerns about the pregnancy, or by encouraging her to attend ANC. Some men also reportedly encourage their pregnant partner to rest, while others encourage her to do gentle exercise to build strength for labour. However other men offer limited support to their pregnant partners:

*When she is pregnant, that’s it, the job is done*. (Expectant Fathers’ FGD, Chimbu Province).

### Attitudes to expectant fathers participating in ANC consultations

Most pregnant mothers, older women and health workers participating in this study were supportive of expectant father’s participating in ANC and highlighted important potential benefits to greater men’s participation, including improved knowledge among expectant fathers of health needs and danger signs during pregnancy, and greater support for pregnant mothers to access health care services.

*We want our husbands to ask the health care workers about our health condition during consultations*. (Pregnant Mothers’ FGD, Southern Highlands Province).

*Mothers are really ready for men to come with them for the antenatal clinic.* (Health Worker KII).

Importantly, however, pregnant mothers participating in FGDs also reported that some women would not want their male partners to accompany them during an ANC consultation, predominantly because women might feel “shy”. Participants reported that women might feel shy if they are seen in public with their partner, or if their partner sees them during the external physical examination or hears them talking to the health worker about topics related to pregnancy or birth.

*Some [pregnant women] do take their husbands to the clinics while others feel shy for their husbands to accompany them.* (Pregnant Mothers’ FGD, Western Highlands Province).

*We don’t want the man to see our tummy so we don’t want our husbands to come to the clinics.* (Pregnant Mothers’ FGD, Western Highlands Province).

Other pregnant mothers reported that expectant fathers should participate in specific parts of ANC consultations, but should not be present during abdominal or pelvic examinations:

*When it is time for pelvic examination the men should not come because later when there is an argument they will say all sort of things. But we want them to be there for all blood tests and check-up so the doctor can advise them too.* (Pregnant Mothers’ FGD, National Capital District).

Expectant fathers participating in FGDs were asked whether many men would participate in an ANC consultation with their pregnant partner if they were invited to do so by a health worker. In most FGDs, participants reported that many fathers would attend ANC with their partner, while others would not, even if invited.

*If we are invited we will go.* (Expectant Fathers’ FGD, Chimbu Province).

*Fathers have different views, some will listen [to the health workers], but some will say that it is the woman’s work.* (Expectant Fathers’ FGD, Western Highlands Province).

This perceived reluctance of some expectant fathers to participate in ANC was generally attributed to the challenges and barriers to men participating in ANC described in the following section.

### Challenges and barriers to male involvement in ANC

#### Sociocultural norms and taboos

Sociocultural norms and taboos were the most commonly reported barrier to expectant fathers participating in ANC. Participants reported that many men believe that MCH, including ANC attendance, is a woman’s responsibility:

*…the main reason is custom that prevent them [men] from attending ANC. Men think that they are superior or the boss of the family so they are not concerned about the health of the mother and the children. They think it’s the job of mothers to look after the children.* (Health Worker KII).

Some participants also spoke of ANC clinics as ‘women’s places’ or for women only:

*Most fathers think antenatal clinical is for mothers only.* (Expectant Fathers’ FGD, Southern Highlands Province).

*In the village there is a hausman [men’s house] and hausmeri [women’s house]. Men don’t go to the women’s house and women don’t go to the men’s house. There is a big respect between men and women. Some think that ANC is a house for women only so men are not allowed to enter.* (Pregnant Mothers’ FGD, Southern Highlands Province).

Norms around appropriate ways for men to behave towards their female partner were often reported in the form of negative community perceptions and gossip about men ‘following’ their partner to the clinic:

*Some men care too much about what others will think of them. [They say] ‘he has never seen a woman before, that’s why he’s following his wife’.* (Expectant Fathers’ FGD, Chimbu Province).

*[If men go to the ANC clinic] people in the community will stare at them and say these men follow their wives around too much.* (Expectant Mothers’ FGD, National Capital District).

These norms relating to fathers’ participating in ANC contributed to men feeling shy, embarrassed or ashamed to attend ANC with their pregnant partner:

*There’s no waiting house for me so I’m ashamed to go inside and I wait outside.* (Expectant Fathers’ FGD, Chimbu Province).

*We encourage them to come but they don’t, a few that come, don’t go inside because they feel shy because too many women look at them. Less than 10% accompany their wives to the ANC.* (Health Worker KII).

While feeling ashamed was a commonly reported barrier to expectant fathers’ participating in ANC, most expectant fathers participating in this study did not explain the reasons why men might feel ashamed to accompany their partner to ANC. However, health workers, pregnant mothers and older women tended to associate men’s feelings of shame or embarrassment with being in the company of large numbers of women (traditionally taboo behaviour in many communities), with observing the health worker examining their pregnant partner’s abdomen, or with having too many children or closely spaced pregnancies.

Shame as a barrier to men participating in ANC was particularly reported by expectant fathers in FGDs in the Highlands Region. In these areas, prevailing gender norms and dominant forms of masculinity may contribute to men feeling too shy or ashamed to publically show support for their pregnant partner:

*I’m a Western Highlander, we have an attitude problem, we are ashamed so we only give money to the woman [to go to the clinic].* (Expectant Fathers’ FGD, Western Highlands Province).

*Young men do care about their partners, it is just that they are shy to show their support in front of their peers.* (Older Men’s FGD, Western Highlands Province).

These community attitudes and the dominant forms of masculinity, particularly in the Highlands, undermine men’s ability to perform caring acts publicly and act as a barrier to men accompanying their partner to ANC.

#### Fear of an HIV test

Some participants in this study spoke of men being reluctant to attend an ANC clinic because they feared having an HIV or STI test. This fear was particularly associated with men who suspected they may be HIV positive or men who had had multiple sexual partners.

*They are scared of being tested for HIV/STIs if they suspect themselves.* (Expectant Fathers’ FGD, Chimbu Province).

*Most men are scared to go to the clinics if they had had multiple sexual partners before their marriage. They are scared of being tested*. (Older Men’s FGD, Chimbu Province).

Participants also highlighted men’s concern that the community will presume that a couple attending ANC together are HIV positive. Health worker practices may be unintentionally reinforcing this assumption; although the majority of health workers supported greater involvement of fathers in ANC, in practice fathers were only actively encouraged to attend if the pregnant mother was found to have an STI or be HIV positive, or to discuss family planning.

*We encourage women to bring partners especially for STI treatment at the same time we provide basic HIV/AIDS information on how it is transmitted and the importance of treatment and prevention*. (Health Worker KII).

*Once the mother come here looking sick like anaemia or the mother have children one after another (like more than five) or when the woman is detected with Sexually Transmitted Infection then we involve the husband on how to go about solving the problem through family planning and counselling.* (Health Worker KII).

#### Poor health worker attitudes

The attitudes of some health workers may further dissuade men from attending clinic services with their partners. Some participants spoke of health workers speaking and behaving in ways that made men feel unwelcome in ANC clinics, and even not allowing men to participate in ANC.

*Nurses are harsh with the man because most men don’t attend the clinic with their wives. The first impression given to them keeps the man from attending the clinic with their wives.* (Older Men’s FGD, National Capital District).

This viewpoint was supported by a health worker, who identified health worker attitudes as a barrier to fathers’ participation in ANC:

*Sometimes health workers have no understanding. They impose their values on fathers…If a man is willing to do this [come to the clinic] we as health workers must comply. Traditionally we are used to seeing women in the clinic, this perception needs to change.* (Health Worker KII).

#### Concurrent relationships

The nature of a couple’s relationship was also reported to influence men’s involvement in ANC. In Southern Highlands Province and Western Highlands Province, men in polygamous relationships were reportedly less likely to accompany their partner to ANC for fear of being perceived as favouring one wife over others, which may cause conflict in the household:

*Men who have many wives don’t go because the community might think he only favours one and not the others.* (Older Men’s FGD, Southern Highlands Province).

In Chimbu and National Capital District, participants spoke more often of men not attending ANC because they worried about being seen by a current or ex-girlfriend:

*Some men don’t want to go to the clinic if they had an affair with a female health worker. Some men don’t want their ex-girlfriends to see them with their wife in case they gossip about them, that’s why they hide.* (Expectant Fathers’ FGD, Chimbu Province).

Some men were also reportedly unlikely to attend ANC with a pregnant partner because they did not wish to make their relationship public, as this would hinder their chances with other partners, or because they were already involved with other women and were not very concerned for or supportive of their pregnant partner.

*When they see another woman, they don’t want to follow their wife to the clinic. The sight of a new woman makes them forget about their wife*. (Older Women’s FGD, Chimbu Province).

#### Time, distance and cost of attending ANC

Limited time, coupled with long waiting times for ANC, was highlighted as a barrier to fathers’ participation in ANC. Long distances to the nearest clinic or lack of money for transport also had a negative impact on men’s participation in clinic services.

*Working husbands don’t have time to come with their wives to the clinic. Those who are unemployed cannot afford the bus to accompany their wives.* (Health Worker KII).

### Enablers that support male involvement in ANC

#### Positive health worker and community attitudes

Enablers to male involvement were closely related to the barriers discussed above. Men were reportedly more willing to attend ANC if the community is supportive of fathers attending clinic and if health workers are friendly and welcoming.

*When the community are happy and compliments a man for bringing his wife to the clinic, it kind of motivates him to take his wife to the clinic.* (Expectant Fathers’ FGD, Chimbu Province).

*Sometime it depends on the health workers. If the health workers are friendly and kind to both the husband and the wife, they both turn up on their clinic days.* (Older Men’s FGD, Chimbu Province).

#### Concerns for the health of their partner or baby

Male FGD participants spoke of expectant fathers being motivated to attend ANC by a sense of shared responsibility for the baby’s health, by a belief that attending clinic together will be good for the baby’s health, or by love for their pregnant partner:

*I accompany my wife [to ANC] because we agreed together to produce the child.* (Expectant Fathers’ FGD, National Capital District).

*Some men are motivated because some couples who frequently go to clinic together produce healthy children and have a healthy family so this motivates them.* (Older Men’s FGD, National Capital District).

Participants noted that many men would be more likely to offer support when his partner is pregnant with their first child than with subsequent pregnancies:

*There are grave concerns for a first born, second born they are not concerned because it is natural.* (Expectant Fathers’ FGD, Western Highlands Province).

Men also spoke of being more likely to attend ANC if their pregnant partner is unwell or diagnosed with HIV or an STI. Similarly, health workers reported that they invite expectant fathers to participate in ANC if the mother is experiencing specific health conditions, such as anaemia or an STI or HIV, or when a couple already have many children in order to discuss family planning.

#### Concerns regarding safety or a woman’s ability to communicate with clinic staff

Expectant fathers are reportedly more likely to participate in ANC if their pregnant partner is illiterate or unable to speak *Tok Pisin* or English to communicate with health workers. Concerns about a woman’s safety on the trip to the clinic may motivate men to accompany their partners to the ANC clinic, although in such cases expectant fathers would not necessarily participate in ANC consultations but rather wait outside the clinic.

### Opportunities for increasing men’s participation in ANC

#### Inviting expectant fathers to attend ANC

Focus group discussion participants were asked to suggest strategies for encouraging fathers to participate in ANC. Individual invitations from health workers to expectant fathers, encouraging them to attend ANC with their pregnant partner was a strategy suggested by both male and female FGD participants. Written invitations would reportedly make expectant fathers feel welcome and ‘special’:

*If we are given an invitation individually, we will feel special and important and cooperate.* (Expectant Fathers’ FGD, Chimbu Province).

One health worker suggested routinely inviting expectant fathers to participate in ANC as a strategy for overcoming men’s fear of community gossip if they participate in ANC:

*People think that it is not good for men to accompany their wife to ANC because of the fear of gossip. Maybe if we make it normal, a routine exercise, I think it will encourage men folk to come with their wives to ANC*. (Health Worker KII).

#### Making facilities welcoming to men

Ensuring all health workers treat expectant fathers in a friendly and respectful manner, and making male health workers available to provide workshops or seminars, were commonly suggested strategies for encouraging men’s participation in ANC.

*Maybe the nurses should be welcoming and open up to the men. If our attitude is good we will bring people in especially in the Antenatal Clinic.* (Health Worker KII).

*Male health workers must encourage the men and give them some special lessons. There must be room provided for men only at the health facilities to give workshop/seminar that will help them to look after their wives and children.* (Pregnant Women’s FGD, Southern Highlands Province).

Many participants suggested adapting facilities to welcome men, including by providing appropriate waiting spaces for men, providing pamphlets and information aimed specifically at men and couples, and providing services such as tea and coffee facilities and entertainment for men (e.g. for example games or videos).

#### Raising community awareness

Older men, older women, and health workers participating in this research reported that increasing community awareness regarding men’s role in supporting the health of their partner and baby will be an important step in increasing male involvement in MCH more generally, and ANC specifically.

*To get men involved in ANC, there should be more awareness about the importance of ANC. It would be good to sit and talk with the men. Also trained health care workers should give special education to the men.* (Health Worker’s KII).

Engaging older people and community leaders to promote and champion greater participation of expectant fathers in ANC was highlighted as a strategy likely to be effective. Integrating antenatal care in outreach health services to provide information sessions to communities was also suggested.

*Develop or find some ways to raise awareness on encouraging men to accompany their wives, emphasizing both couples coming to the clinic. Health staff at the clinic should have some time available to do mobile clinic at the village to give education on involving men because most of the men are not aware [that they can attend ANC].* (Older Women’s FGD, Western Highlands Province).

#### Compulsory attendance and incentives

Some participants in FGDs and KIIs suggested making ANC attendance compulsory for expectant fathers and penalizing pregnant women by refusing service or levying a fee if they did not bring their partner to ANC.

*If the wife turns up at the clinic on her own, the service fee should be increased but if both turn up it should be reduced. Service fee should be controlled in a way that both partners will visit the clinic.* (Older men’s FGD Chimbu Province).

*In order to bring the men to the clinic we should engage the leaders to tell their people that both men and women should go to antenatal clinic because of the prevalence of the HIV and we will make it compulsory that the pregnant mother is seen and treated as long as she comes with the husband. If not she will not be treated, it will be compulsory.* (Health Worker’s KII).

As noted above, however, some female participants reported a preference for attending ANC alone. Participants suggesting compulsory ANC attendance for male partners did not reflect on the potential negative consequences of such an approach.

## Discussion

This qualitative research paper has identified key barriers and enablers to expectant fathers’ participation in ANC consultations in PNG, explored attitudes to fathers participating in ANC, and identified opportunities to encourage fathers to accompany their partner to ANC. In general, women and health workers consulted in this study were supportive of expectant fathers participating in ANC consultations, and these participants highlighted a range of benefits that greater male involvement could have for women and children.

Importantly, however, this study also found that some women prefer to attend ANC alone or prefer male partners to be present during ANC counselling but not during physical examinations. This finding is consistent with previous research from PNG and other settings, which has shown that attending ANC alone can offer a valued opportunity to travel unaccompanied, network with other women, and/or privately seek health advice or services [41, 60]. In seeking to involve expectant fathers in ANC, policy makers and health workers must ensure that women are able to decide whether and when their partner joins them in ANC consultations. A range of strategies exist for engaging fathers without compromising women’s privacy or autonomy, including: inviting each expectant father to attend ANC via his pregnant partner, explicitly allowing her to decide whether to pass on this invitation; allocating time at the start of a consultation to talk to a woman alone, before asking if she would like her male partner to join the consultation; allowing women to bring their male partners to ANC counselling, but ensuring that physical examinations and potentially sensitive topics are discussed in private; or seeking to routinely involve expectant fathers in only the first or second visit. While some participants in this study suggested making men’s attendance at ANC compulsory or providing disincentives for women attending ANC without a partner, such approaches are not recommended as they can stigmatise single or unaccompanied women and dissuade these women from accessing ANC [30, 61]. Regardless of the strategy employed to engage fathers, pre-testing messages and strategies with both men and women will be important in maximising the benefits of engaging men in MCH while minimising potential risks such as loss of women’s autonomy in health decision-making [19].

The finding from this study, and other research in PNG [41, 62], that some expectant fathers wait outside the clinic while their pregnant partner attends an ANC consultation highlights an opportunity to engage men in ANC consultations or other health education initiatives. Health workers should consider routinely asking women if their partner is waiting nearby and if they would like him to join the consultation. Community members, including expectant fathers, involved in this study believed that expectant fathers would respond positively to a written or verbal invitation to participate in ANC. This finding is in line with international research that has shown that even in contexts where ANC is considered ‘women’s business’, inviting expectant fathers to attend ANC if their pregnant partner would like them to can make men feel more welcome at ANC [63], and can increase expectant father’s participation in ANC [24, 25, 35, 58, 64, 65], particularly when invitations are tailored to local health concerns [64]. Routinely inviting fathers to participate in one or more ANC consultations (with their pregnant partner’s consent), rather than the current practice of inviting fathers to ANC only when their partner tests positive to HIV or an STI, or is experiencing a serious health issue, may also reduce stigma and gossip associated with men’s attendance at ANC, thereby reducing barriers to couples attendance at ANC in the future.

Our finding that health worker attitudes and capacity are a barrier to expectant fathers’ participation in ANC is similar to research findings in other contexts [31, 35, 41, 42, 44] and highlights the need to ensure that nursing and midwifery education and in-service training include a focus on respectful, family-centered care and couple counseling skills. Training health workers to engage fathers and provide quality couple counseling has been effective in increasing men’s participation in ANC services [24, 25] and increasing health worker job satisfaction [24] in other settings and should be trialed in PNG.

The perception reported by study participants that ANC clinics are women’s places is a finding echoed in the global literature [31–35, 40, 41, 66–68]. In our study clinics, as in most public clinics in PNG, women are unable to make appointments for ANC and instead go to the clinic on designated ANC days, often waiting for long periods before being seen. Further, clinics often lack privacy for women receiving counselling or examinations. These factors contribute to men feeling embarrassed and intimidated when accompanying their pregnant partner, and may make other women seeking ANC uncomfortable. Existing and future ANC clinics can be made more father- and couple-friendly by ensuring consultation spaces afford adequate privacy, providing specific waiting areas for men and couples, having separate entrances for men, or displaying posters, magazines or educational DVDs that specifically target and depict men. The existing layout and resources available to clinics in PNG means that some of these changes are likely to be immediately feasible in at least some clinics but not others, underscoring the need for a range of strategies that are selected or adapted based on the local community and resources available. Notably, many suggested intervention to make clinics more ‘father friendly’ – such as ensuring the physical environment affords adequate privacy, providing alternative opening hours and ensuring staff are supported to provide quality couple counselling and respectful care – are also likely to make existing services more acceptable to pregnant women and adolescents.

Other suggested changes such as providing games for men waiting at ANC are likely to be less attractive to health staff and policymakers, due to the expense and the potential to distract men from information and services provided at the clinic. However health workers may be able to harness this expressed preference for entertainment in order to increase men’s health-related knowledge; program experience indicates that facilitated, game-based health education for men can be feasible and highly acceptable in PNG [69] and could be usefully integrated into group antenatal education for expectant fathers [70].

Community awareness raising was frequently highlighted by participants as critical to increasing expectant fathers’ participation in ANC and has been shown internationally to be effective in increasing men’s engagement in MCH [54, 71]. Communication strategies, such as mass and social media and interpersonal communication strategies, should seek to provide information and stimulate discussion about the benefits of expectant fathers participating in ANC, while specifically addressing negative community attitudes about men ‘following’ their partner to ANC. Communication interventions through community groups and institutions, targeting both younger and older people, may also be an appropriate strategy to reach men with information about MCH while barriers to men’s full participation in ANC are addressed. Engaging men in settings where they commonly congregate has long been a recommendation of the male engagement literature [72, 73] and research indicates that men prefer to be engaged in places where they meet socially [74].

Participants in this study reported that prevalent ideas regarding masculinity, a man’s role versus women’s responsibilities, and men’s spaces versus women’s places, tend to limit male involvement in ANC. However, participants also reported divergent community attitudes towards expectant fathers participating in ANC, noting that while some community members criticise and gossip about expectant fathers attending ANC with their pregnant partner, other community members are pleased and supportive. Identifying and supporting strong role models to champion the role of fathers in MCH, including the importance of expectant fathers participating in ANC, may increase the acceptability of men attending ANC and has been shown to be an effective strategy in engaging men in other settings [75, 76].

Our findings suggest that some expectant fathers, and indeed pregnant women, will not choose to participate in ANC consultations together, even if given the option to do so. Promising findings from the international literature suggest that men-only group education or one-on-one peer-education can break down barriers to men’s participation in MCH and encourage fathers to take a more active, positive role in MCH [23, 24, 76–78]. As many clinics in PNG only provide antenatal care on specific days and at specific times, and given our finding that some expectant fathers wait outside the clinic while their partner receives ANC, men-only group antenatal education or one-on-one peer education may be a viable option to reach these men. Group education in particular is relatively low cost and has proven effective in improving care-seeking and health-related behaviours [23, 24, 57, 78]. Program experience also suggests that men’s group antenatal education is feasible and acceptable in PNG [70].

The finding that men with multiple sexual partners, either in the form of extramarital partners or multiple wives, may be less likely to participate in ANC services and may avoid clinics for fear of an STI or HIV test is particularly concerning. Some 13% of pregnant mothers involved in this study reported being in a polygamous relationship, suggesting that a substantial proportion of women and children are likely to be impacted if we fail to reach polygamous men with information and services. Further research is needed on the most suitable approach to engaging this population group in antenatal education, but interventions such as peer-to-peer counselling or men’s group health education may be appropriate.

This study has some important limitations. Due to the short timeframe available to collect data between commencement of this study and the 2012 General Elections,^1^ participants were largely recruited through clinics and public announcements in churches and community meetings, which may have biased participation towards those accessing clinic services, attending church or with a pre-existing interest in health. In addition, voice recordings of focus groups and interviews were of poor quality due to excessive background noise and softly spoken participants (as described earlier). Ultimately written records compiled from detailed data collector notes, supplemented with transcribed voice recordings where possible, were used for analysis. Due to budget and time constraints, analysis and findings were checked with provincial data collection teams but not with participants. Finally, data for this study were collected in National Capital District and the Highlands Region of PNG. Social and cultural diversity across PNG means that the findings of this study may not be applicable to other geographical locations, such as coastal or island regions.

## Conclusion

Expectant fathers in PNG face considerable barriers to participating in ANC with their pregnant partners, including sociocultural norms and taboos, inappropriate clinic infrastructure and poor health workers attitudes. Although many men accompany their pregnant partner to the ANC clinic, few participate in ANC consultations. Findings suggest, however, that most pregnant women and health workers support fathers participating in ANC and that at least some expectant fathers would attend ANC consultations if invited to do so.

This study has also identified strategies for increasing expectant fathers’ participation in ANC, with implications for program planners seeking to encourage men to take an active, positive role in supporting maternal and child health. Interventions such as explicitly inviting expectant fathers to participate in ANC services, if their pregnant partner would like them to, and ensuring that health workers have the skills to engage men and provide quality couple ANC counselling will be important in increasing men’s participation in ANC. Interventions to make clinic spaces more welcoming to expectant fathers – such as providing posters and pamphlets depicting and targeting fathers, or providing men’s or couple’s waiting spaces – may also be feasible in many clinics. Community awareness raising interventions will also be needed to build community support for expectant fathers’ participation in ANC. Other promising strategies, such as men-only group antenatal education, should be considered to reach men unable or unwilling to attend ANC with their partner.

## Additional files

Additional file 1: Sample Focus Group Discussion Guide: Pregnant women. Sample questions used by facilitators to guide discussions with pregnant women. (DOCX 172 kb)

Additional file 2: Sample Focus Group Discussion Guide: Expectant fathers. Sample questions used by facilitators to guide discussions with expectant fathers. (DOCX 158 kb)

Additional file 3: Sample Focus Group Discussion Guide: Older women. Sample questions used by facilitators to guide discussions with older women. (DOCX 126 kb)

Additional file 4: Sample Focus Group Discussion Guide: Older men. Sample questions used by facilitators to guide discussions with older men. (DOCX 126 kb)

Additional file 5: Sample Key Informant Interview Guide: Health Workers. Sample questions used by facilitators to guide discussions with health workers. (DOCX 128 kb)

## Abbreviations

ANC: Antenatal care
FGD: Focus Group Discussion
HIV: Human immunodeficiency virus
KII: Key Informant Interview
MCH: Maternal and Child Health
PNG: Papua New Guinea
PPTCT: Prevention of parent-to-child transmission of HIV
STI: Sexually transmitted infection

## Footnotes

1 Election periods in PNG are generally associated with heightened population movement as people move to their home areas to vote, and greater instability and insecurity.
