# Supplementary material for: Expectant fathers’ participation in antenatal care services in Papua New Guinea: a qualitative inquiry
Source: BMC Pregnancy Childbirth. 2018 May 8;18:138. doi: 10.1186/s12884-018-1759-4 (PMC5941321; doi:10.1186/s12884-018-1759-4)
Supplement: Supplementary file 1 — Sample Focus Group Discussion Guide: Pregnant women. Sample questions used by facilitators to guide discussions with pregnant women. (DOCX 172 kb) [file 12884_2018_1759_MOESM1_ESM.docx]

**Sample Focus Group Discussion Guide: Pregnant Women**

**Health during pregnancy**

*Objectives:*

- *Explore participants’ awareness of the importance of early antenatal care for pregnant women (starting in first 3 months).*
- *Understand attitudes to men’s involvement in antenatal care and sexual and reproductive health services, and explore perceived barriers and enablers to men’s involvement.*

1. Do pregnant women usually talk to anyone else about looking after their own health and their baby’s health during pregnancy?

*(Who do pregnant women talk to? Why do they talk to those people?)*

1. Do pregnant women talk to their husbands about pregnancy and health during pregnancy?

- What makes it difficult for some women to talk to their husband about health during pregnancy?
- What would make it easier for pregnant women to talk to their partner about health during pregnancy?

*(e.g. Does it depend on the relationship between the couple? Does it depend how many children the couple have had? Would it be easier if pregnant women had some information material to give their husband about health during pregnancy?)*

1. Do pregnant women think it is important to go to the antenatal clinic?

- What are the good things about the antenatal clinic?
- When during pregnancy should a woman first go to the antenatal clinic?
- When women do go to the antenatal clinic, what are the things they don’t like?

1. What reasons might stop some pregnant women going to the antenatal clinic?

*(e.g. no PMV money, husband doesn’t support it, worried nurses will be angry about something?)*

1. What do men do to support their pregnant wives?

- What would pregnant women like their husbands to do to support them?

1. Do community leaders ever encourage men to support their pregnant wives?

- If a man is not supporting his pregnant wife in any way, what would community leaders think or do?

1. Do many men go to the to antenatal clinic? *(Accompany wife and wait? Go in for baby check-up?)*

- If some men go to the antenatal clinic, why do they go?
- Why don’t some men go to the antenatal clinic?
- What would the community think if a man went to the antenatal clinic with his wife?

1. For men who don’t go to the antenatal clinic, how do they get information about their pregnant wife’s health?
2. Would most women like their husbands to come to the antenatal clinic with them?

- Which parts of the consultation would women like men to come to or not like men to come to?

*(e.g would women like their husbands to talk to the health worker but not to be there during physical examination? )*

1. If health workers invite men to come to the antenatal clinic, would many men accept the invitation and come along?

*(If it’s clear that men can get information about health pregnancy and HIV/STI services and information that will protect their own health and their family’s health?)*

1. Is there anything that could make it easier for men to go to the antenatal clinic with their pregnant wives?

*(e.g. Does it depend on the wife’s attitude or the health workers attitude? Are there any things the antenatal clinic could do to make men feel welcome?)*

**Sex during pregnancy**

*Objectives:*

- *Understand behaviours that put pregnant/breastfeeding women at risk of STIs and HIV: extramarital sexual behaviours, condom use, couple communication regarding HIV, and awareness of STI/HIV risks to unborn babies.*

Now we will talk about sex during pregnancy in Papua New Guinea.

1. Do couples in your community generally have sex during pregnancy?
2. Are there any dangers to having sex during pregnancy or after delivery?

- If yes, what are the dangers?
- When during pregnancy/after delivery are they a concern?
- If couples stop having sex during pregnancy, when do they start having sex again?
- Does the timing vary from one couple to another, and one pregnancy to another?

1. If couples stop having sex during pregnancy, how does this affect the relationship between the couple?

*(Are men still supportive of their partner?)*

1. Where do women get information about sex during pregnancy? *(e.g. TV, friends, relatives, health workers?)*

- Do women feel comfortable asking a health worker about sex during pregnancy?

*Using 10 seed technique…*

*(Note: if men in the community commonly have more than one wife, you should ask how common multiple wives are and how many wives a man usually has. The ‘other women’ in the picture below should include ‘other wives’)*

1. If you think about Papua New Guinea men in general, how many do you think have sex with other women while their wife is pregnant or in the first few weeks after the baby’s arrival?


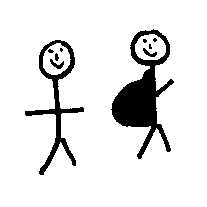


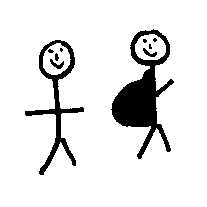


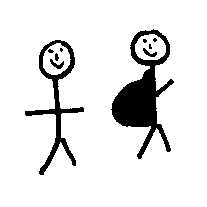


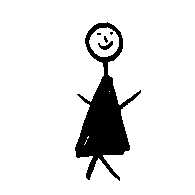


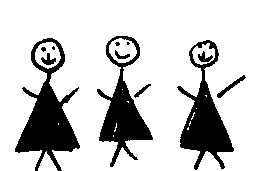


These men only have sex with their pregnant wife

These men would have sex with one other woman

These men would have sex with two or more other women

1. If you think about pregnant women in Papua New Guinea, how many do you think would have sex with other men when she is pregnant or in the first few weeks after delivery?


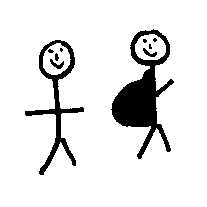


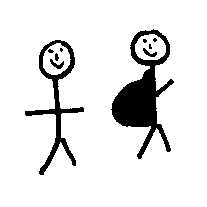


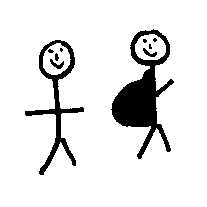


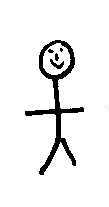


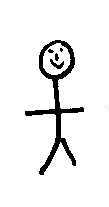

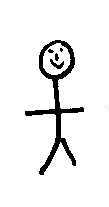

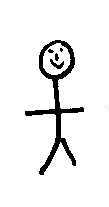


These pregnant women would only have sex with their husband

These pregnant women would have sex with another man

These pregnant women would have sex with two or more other men

1. Do you think many women use condoms when they have sex with someone other than their husband?
2. What problems might result when an expectant father or a pregnant woman has unprotected sex with someone else?

*(e.g. The baby might get HIV or be born with syphilis; Violence/anger??)*

**STIs and HIV**

*Objectives:*

- *Understand knowledge and attitudes to STIs and HIV prevention, testing and couple counselling.*
- *Understand attitudes towards PLHIV providing health services.*

1. Do long-term couples talk about HIV prevention?
2. Do women talk about condom use with their husbands or long-term sexual partners?

- If no, what makes it difficult to talk about using condoms within couples?
- What could make it easier for couples to talk about condom use?

*(e.g. is it easier if couples have written information about condom use? Is it easier for couples who have been in a relationship a long time?)*

1. Are there any factors that might stop a woman from having an HIV test (if they think there is a chance they have HIV)?
2. Do women who have tested for HIV tell their husbands about the HIV test result? (*Why/why not?)*

- What are the risks of talking to the partner about test results? *(e.g. Is there a risk of violence?)*
- What would make it easier for couples to talk about their HIV test results?

1. Is it a good idea to counsel and test pregnant women for HIV together with their husbands? (*Why/why not?)*

- What are the risks of testing couples together? *(e.g. Is there a risk of violence?)*

1. If a pregnant woman gets infected with HIV during pregnancy, what can happen to the baby?

- *(If they believe there is a risk to the baby)* Is there anything a pregnant woman can do to reduce the risk of passing HIV to her baby?

1. If a pregnant woman gets infected with an STI during pregnancy, what can happen to the baby? *(e.g. syphilis)*

- *(If they believe there is a risk to the baby)* Is there anything a pregnant woman can do to reduce the risk of passing the STI to her baby?

1. Some health centres are planning to train women who are HIV positive to provide HIV counselling, testing and support services in the clinic. What would the community think about this?

*(Would people still attend the antenatal clinic if someone with HIV was working at the clinic? Why?)*

**Feeding babies**

*Objectives:*

- *Explore attitudes/practices regarding breastfeeding, to help design effective PPTCT messages.*

1. Can a mother with HIV breastfeed her baby safely?
2. Do fathers affect baby feeding in any way?

*(e.g. Do fathers make decisions about how long a baby is fed breast milk or when other foods are given?)*
