# Supplementary material for: Expectant fathers’ participation in antenatal care services in Papua New Guinea: a qualitative inquiry
Source: BMC Pregnancy Childbirth. 2018 May 8;18:138. doi: 10.1186/s12884-018-1759-4 (PMC5941321; doi:10.1186/s12884-018-1759-4)
Supplement: Supplementary file 2 — Sample Focus Group Discussion Guide: Expectant fathers. Sample questions used by facilitators to guide discussions with expectant fathers. (DOCX 158 kb) [file 12884_2018_1759_MOESM2_ESM.docx]

**Sample Focus Group Discussion Guide: Expectant Fathers**

**Health during pregnancy**

*Objectives:*

- *Explore participants’ awareness of the importance of early antenatal care for pregnant women (starting in first 3 months).*
- *Understand attitudes to men’s involvement in antenatal care and sexual and reproductive health services, and explore perceived barriers and enablers to men’s involvement.*

1. Do men talk to their wives about pregnancy and health during pregnancy?

- If they do not talk to their wives, who do they talk to?
- What makes it difficult for some men to talk to their wives about health during pregnancy?
- What would make it easier for men to talk to their wives about health during pregnancy?

1. Do men think antenatal clinic visits are a good idea for pregnant women?

- Why/why not?
- When during pregnancy should a woman first go to the antenatal clinic?
- Are there any reasons why some men don’t like their pregnant wives going to the antenatal clinic?

1. What do men do to support their wife during pregnancy?

- What should a man do to support his wife during pregnancy?

1. Do community leaders ever encourage men to support their pregnant wives?

- If a man is not supporting his pregnant wife in any way, what would community leaders think or do?

1. Do many men go to the to antenatal clinic? *(Accompany wife and wait? Go in for baby check-up?)*

- If some men go to the antenatal clinic, why do they go?
- Why don’t some men go to the antenatal clinic?
- What would the community think if a man went to the antenatal clinic with his wife?

1. If men do not go to the antenatal clinic, how do they find out information about their pregnant wife’s health?
2. If health workers invite men to come to the antenatal clinic, would many men accept the invitation and come along?

*(If it’s clear that men can get information about health pregnancy and HIV/STI services and information that will protect their own health and their family’s health?)*

1. If STI testing or treatment or HIV testing were provided for men in the antenatal clinic, what concerns would men have about getting these services together with their pregnant wife?
2. Are there any services that men would like the antenatal clinic to offer for men or for pregnant women?
3. Is there anything that could make it easier for men to go to the antenatal clinic with their pregnant wives?

*(e.g. Does it depend on the wife’s attitude or the health workers attitude? Are there any things the antenatal clinic could do to make men feel welcome?)*

1. What sort of information would men like to know so they can help their pregnant wife and children stay healthy?
2. How should we get information to men about health during pregnancy?

- Who would be the best people to provide this information? *(health worker, leaders?)*
- Where should this take place? *(workplace, mens haus, antenatal clinic?)*
- How would men like to receive this information? *(pamphlets, group talks, individual talks?)*

**Sex during pregnancy**

*Objectives:*

- *Understand behaviours that put pregnant/breastfeeding women at risk of STIs and HIV: extramarital sexual behaviours, condom use, couple communication regarding HIV, and awareness of STI/HIV risks to unborn babies.*

Now we will talk about sex during pregnancy in Papua New Guinea.

1. Do couples generally have sex when the wife is pregnant?
2. Are there are any dangers to having sex during pregnancy or after delivery?

- If so, what are these dangers?
- When during pregnancy/after delivery are they a concern?
- If couples stop having sex during pregnancy, when do they start having sex again?
- Does the timing vary from one couple to another, and one pregnancy to another?

1. If couples stop having sex during pregnancy, how does this affect the relationship between the couple?

*(Are men still supportive of their partner?)*

1. If a woman feels uncomfortable having sex during pregnancy or afterwards, what do husbands do?

*(Have sex with her anyway? Have sex without intercourse? Masturbate? Have sex with someone else?)*

1. Where do men get information about sex and pregnancy? *(e.g. TV, friends, relatives, health workers?)*

- Do men feel comfortable asking a health worker about sex during pregnancy?

*Using the 10 seed technique…*

*(Note: if men in the community commonly have more than one wife, you should ask how common multiples wives are and how many wives a man usually has. The ‘other women’ in the diagram below should include ‘other wives’ )*

1. If you think about Papua New Guinea men in general, how many do you think have sex with other women while their wife is pregnant or in the first few weeks after the baby’s arrival?


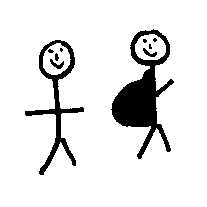


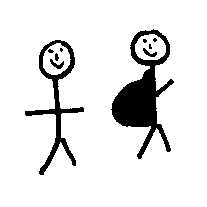


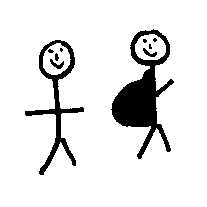


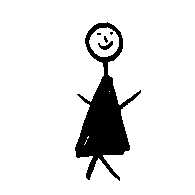


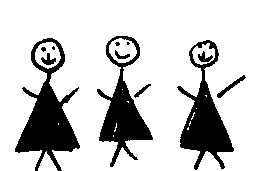


These men only have sex with their pregnant wife/partner

These men would have sex with one other woman

These men would have sex with two or more other women

1. Do you think many men use condoms when they have sex with someone other that their pregnant wife?
2. What problems might result when an expectant father has unprotected sex with someone other than his pregnant wife?

*(e.g. The baby might get HIV or be born with syphilis. Violence/anger??)*

**STIs and HIV**

*Objectives:*

- *Understand knowledge and attitudes to STIs and HIV prevention, testing and couple counselling.*
- *Understand attitudes towards PLHIV providing health services.*

1. Do long-term couples talk about HIV prevention?
2. Do men talk about condom use with their wives or long time sexual partners?

- If no, what makes it difficult to talk about using condoms within relationships?
- What could make it easy for couples to talk about condom use?

*(e.g. is it easier if couples have written information about condom use? Is it easier for couples who have been in a relationship a long time?)*

1. What would a man in Papua New Guinea do if he suspected he may have an STI?

- Would he get tested? *(Where would he get tested?)*
- Would he seek treatment? *(At a pharmacy? Clinic? Why would he seek treatment at this place?)*
- Would he tell his wife? *(Why/why not?)*

1. Are there any factors that might stop a man from having an HIV test (if they think there is a chance they have HIV)?
2. Do men who have tested for HIV tell their wives about the HIV test result?

- What could make it easier for couples to talk about their HIV test results?

1. Do you think it is a good idea to counsel and test pregnant women for HIV together with their husbands?

- Why/why not?
- What are the risks of testing couples together? *(e.g. Is there a risk of violence?)*

1. If a woman gets infected with HIV during pregnancy, what can happen to the baby?

- *(If they believe there is a risk to the baby)* Is there anything a pregnant woman can do to reduce the risk of her baby being born with HIV?

1. If a woman gets infected with an STI during pregnancy, what can happen to the baby? *(e.g. syphilis)*

- *(If they believe there is a risk to the baby)* Is there anything a pregnant woman can do to reduce the risk of passing the STI on to her baby?

1. Some health centres are planning to train and employ women who are HIV positive to provide HIV counselling, testing and support services in the antenatal clinic. What would the community think about this?

*(e.g. Would people still attend the antenatal clinic if someone with HIV was working at the clinic? Why?)*

**Feeding babies**

*Objectives:*

- Understand attitudes and practices relating to breastfeeding, to help design effective PPTCT messages.

1. Some HIV positive mothers may need to take HIV medicine to protect their baby from HIV. This medicine needs to be taken every day while the mother is pregnant or breastfeeding. In general, do you think husbands would support their HIV positive wives to take this medicine?

*(Why/why not?)*

1. Health workers tell all mothers to feed their babies only breast milk for the first six months. Do men play any role in supporting their wives to feed babies?
