# Supplementary material for: Expectant fathers’ participation in antenatal care services in Papua New Guinea: a qualitative inquiry
Source: BMC Pregnancy Childbirth. 2018 May 8;18:138. doi: 10.1186/s12884-018-1759-4 (PMC5941321; doi:10.1186/s12884-018-1759-4)
Supplement: Supplementary file 3 — Sample Focus Group Discussion Guide: Older women. Sample questions used by facilitators to guide discussions with older women. (DOCX 126 kb) [file 12884_2018_1759_MOESM3_ESM.docx]

**Sample Focus Group Discussion Guide: Older Women**

**Health during pregnancy**

*Objectives:*

- *Explore awareness of the importance of early antenatal care for pregnant women (starting in first 3 months).*
- *Understand attitudes to male involvement in antenatal care and sexual and reproductive health services, and explore perceived barriers and enablers to their involvement.*

1. Do younger women ask older women like you for advice on health during pregnancy?

- Who asks for information?
- What kind of information do you give?

1. What do pregnant women think about attending antenatal clinic?

- What are the good things about antenatal clinic?
- When during pregnancy should a woman first go to the antenatal clinic?
- Are there any things women don’t like when they go to the antenatal clinic?

1. Are there reasons that prevent pregnant women from going to the antenatal clinic?
2. What do men do to support their pregnant wives?
3. Do community leaders ever encourage men to support their pregnant wives?

- If a man is not supporting his pregnant wife in any way, what would community leaders think or do?

1. Do many men go to the antenatal clinic? *(Accompany wife and wait? Go in for baby check-up?)*

- Why don’t some men go to the antenatal clinic?
- Would men like to go to the antenatal clinic with their pregnant wife and receive services and information for his own health and the health of his wife and baby?

1. Would women like their husbands to come to the antenatal clinic with them?
2. Is there anything that could make it easier for men to go to the antenatal clinic with their pregnant wives?

**Sex during pregnancy**

*Objectives:*

- *Understand knowledge, beliefs and behaviours regarding sex during pregnancy.*

Now we will talk about beliefs about sex during pregnancy in Papua New Guinea.

1. Do younger women ask older women like you for advice on whether sex during pregnancy is safe for the mother and baby?

- What kind of questions do younger women ask?

1. Are there any dangers to having sex during pregnancy or after delivery?

- What are the dangers?
- When during pregnancy/ after delivery are they a concern?
- If couples stop having sex during pregnancy, when do they start having sex again?
- Does the timing vary from one couple to another, or from one pregnancy to another?

1. Where do women get information about sex during pregnancy and after delivery?

- Who gives this information?
- Do women feel comfortable asking a health worker about sex during pregnancy?
- How would women feel if the doctor or health worker gave you this information?

1. If women don’t want to have sex during pregnancy or after delivery, what do husbands do if they feel like having sex?

*(e.g have sex with her anyway? have sex without intercourse? masturbate? have sex with someone else?)*

**Feeding babies**

*Objectives:*

- *Understand attitudes and practices relating to breastfeeding, to help design effective PPTCT messages.*

1. Health workers tell all mothers to feed their babies only breast milk for the first six months. Is this difficult for some mothers?

- If so, why is it difficult and what kinds of worries do women have about feeding babies?

1. Do fathers affect baby feeding in any way?

*(e.g. Do fathers make decisions about how long a baby is fed breast milk or when other foods are given?)*

1. This is the end of our questions, is there anything else you want to say about any of the topics we have talked about today?
