# Supplementary material for: Expectant fathers’ participation in antenatal care services in Papua New Guinea: a qualitative inquiry
Source: BMC Pregnancy Childbirth. 2018 May 8;18:138. doi: 10.1186/s12884-018-1759-4 (PMC5941321; doi:10.1186/s12884-018-1759-4)
Supplement: Supplementary file 4 — Sample Focus Group Discussion Guide: Older men. Sample questions used by facilitators to guide discussions with older men. (DOCX 126 kb) [file 12884_2018_1759_MOESM4_ESM.docx]

**Sample Focus Group Discussion Guide: Older Men**

**Health during pregnancy**

*Objectives:*

- *Explore awareness of the importance of early antenatal care for pregnant women (starting in first 3 months).*
- *Understand attitudes to men’s involvement in antenatal care and sexual and reproductive health services, and explore perceived barriers and enablers to their involvement.*

1. Do younger men ask older men like you for advice on health during pregnancy?

- Who asks you for information?
- What kind of information do you give?

1. What do men think about pregnant women attending antenatal clinic?

- What are the good things about antenatal clinic?
- When during pregnancy should a woman first go to the antenatal clinic?
- Are there any reasons why some men don’t like their pregnant wives going to the antenatal clinic?

1. What do men do to support their pregnant wives?

- What should a man do to support his wife during pregnancy?

1. Do community leaders ever encourage men to support their pregnant wives?

- If a man is not supporting his pregnant wife in any way, what would community leaders think or do?

1. Do many men go to the to antenatal clinic? *(Accompany wife and wait? Go in for baby check-up?)*

- If some men go to the antenatal clinic, why do they go?
- Why don’t some men go to the antenatal clinic?
- Would more men like to go to the antenatal clinic with their pregnant wife?

1. Is there anything that could make it easier for men to go to the antenatal clinic with their pregnant wives?
2. Are there any services that men would like the antenatal clinic to offer for men or for pregnant women?
3. Where do men get information about health during pregnancy?
4. What sort of information would men like to know so they can help their pregnant wife and children stay healthy?
5. How should we get information to men about their wives health during pregnancy?

- Who would be the best people to provide this information? (health worker, leaders?)
- Where should this take place? *(workplace, menshaus, antenatal clinic?)*
- How would men like to receive this information? *(pamphlets, group discussions, individual talks?)*

1. What would men like to know about feeding babies when they are small (less than 6 months)?

**Sex during pregnancy**

***Objectives:***

- ***Understand knowledge, beliefs and behaviours regarding sex during pregnancy.***

Now we will talk about beliefs about sex during pregnancy in Papua New Guinea.

1. Do younger men ask older men like you for advice on whether sex during pregnancy is safe for the mother and baby?

- What kind of questions do younger men ask?

1. Are there any dangers to having sex during pregnancy or after delivery?

- What are the dangers?
- When during pregnancy/after delivery are they a concern?
- If couples stop having sex during pregnancy, when do they start having sex again?
- Does the timing vary from one couple to another, and one pregnancy to another?

1. Where do men get information about sex during pregnancy and after delivery?

- Who gives this information?
- Do men feel comfortable asking a health worker about sex during pregnancy? How would you feel if the doctor or health worker gave you this information?

1. If women don’t want to have sex during pregnancy or after delivery, what do husbands do if they feel like having sex?

*(Have sex with her anyway? Have sex without intercourse? Masturbate? Have sex with someone else?)*

1. This is the end of our questions, is there anything else you want to say about any of the topics we have talked about today?
