# Supplementary material for: Expectant fathers’ participation in antenatal care services in Papua New Guinea: a qualitative inquiry
Source: BMC Pregnancy Childbirth. 2018 May 8;18:138. doi: 10.1186/s12884-018-1759-4 (PMC5941321; doi:10.1186/s12884-018-1759-4)
Supplement: Supplementary file 5 — Sample Key Informant Interview Guide: Health Workers. Sample questions used by facilitators to guide discussions with health workers. (DOCX 128 kb) [file 12884_2018_1759_MOESM5_ESM.docx]

**Sample Key Informant Interview Guide: Health Workers**

**Male involvement in antenatal care**

*Objectives:*

- *Understand health workers’ attitudes to male involvement in antenatal care.*

1. In your experience of providing services to pregnant women, are there any situations when it is really important to talk to a woman’s husband? *(What situations?)*
2. Do many men come along with their wives to the antenatal clinic?

- Roughly what percentage of women do you think come to the clinic with their husband?

1. Do you think it’s a good idea for men to attend the antenatal clinic along with their pregnant wives?

- What are the benefits of men coming to the antenatal clinic?
- Are there any disadvantages or risks associated with men coming to the antenatal clinic? *(If so, what?)*

1. Health centres recommend four antenatal care visits for pregnant women. How many times do you think a man should come with his pregnant wife?

- Which antenatal visit should the man attend? *(First? Second? Third? Fourth?)*

1. When men do come to the antenatal clinic, what services or information do you provide them?

- What services or information do you think we should offer to men who come to the antenatal clinic?

1. What are some of the reasons that prevent men coming to the antenatal clinic with their wives?
2. Are there any factors that make it easier for some men to come to the antenatal clinic with their wife?
3. What do you think we could do to get men more involved in the antenatal clinic?

*(e.g. special training for health workers, particular hours for couples to attend the antenatal clinic?)*

- What changes would be required at the facility so health workers can include expectant fathers?

1. What do you think about inviting men along to their child’s first immunization visit?

- Would this be a good time to teach men about health for children, mothers and for men?

**Sex and pregnancy, STIs and HIV**

*Objectives:*

- *Explore knowledge about sex and the risk of STIs and HIV during pregnancy/breastfeeding.*
- *Explore attitudes to HIV; including people living with HIV, testing and counselling.*

1. Do women or men ever ask you about whether sex during pregnancy and breastfeeding is safe for the baby and the mother?

- *[If yes]* What kind of advice do you give them?

1. Do you think there are any dangers to having sex during pregnancy (and breastfeeding)?

- If so, what are these dangers and at what times during pregnancy/after delivery are they a concern?
- What kind of things or which people have influenced your beliefs on this topic?

*(e.g. religion, your parents, community attitudes, medical textbooks, lecturers or others)*

1. If women or men do not ask about sex during pregnancy and breastfeeding, are you able to raise the topic during the antenatal clinic visit?

- If so, how?
- If not, what prevents you from raising the issue of sex during pregnancy?
- Do you feel comfortable talking about this issue?

1. What would help you talk about sex during pregnancy and breastfeeding with your antenatal clients?
2. Do you talk to pregnant women about the risks of STIs and HIV during pregnancy or breastfeeding?

- If yes, what do you tell them about the risks and what kind of advice do you give them?
- If no, what would help you to talk about this issue?

1. What might stop a woman from having an HIV test (when she is pregnant or any other time)?
2. Do women who receive HIV tests generally talk to their partner about their results?

- If not, why not?
- What could we do to make it easier for women to tell their husband about their HIV status?

1. What kinds of training and support have health workers received to help them counsel and test pregnant mothers and their husbands for HIV?
2. What kind of support or resources could help health workers to counsel or test pregnant women and their partners for HIV?

*(e.g. Staffing? Training? Manuals? Better privacy? Better referral networks?)*

1. Do health workers do anything to follow-up women who have had an HIV test but don’t come back for results?
2. Do health workers do anything to follow-up with women who receive a positive HIV test result but don’t return for treatment? (Or start treatment and then don’t come back?)
3. If a man or woman tests positive to HIV, do you refer them on to any community organisations or other support services?
4. Some HIV positive mothers may need to take antiretroviral medicine every day when they are pregnant and breastfeeding to protect the baby from HIV. Is it difficult for some women to take this medicine every day?

- Why/why not?
- What do you think would make it easier for women to take this medicine every day?

1. Is it important for HIV positive women to deliver their babies in a health facility?

- Why/why not?

1. What would you think about women who are HIV positive who are trained to work on HIV working in a clinic to support other women through the process of HIV testing and treatment?
